# Substrate Binding Mode and Its Implication on Drug Design for Botulinum Neurotoxin A

**DOI:** 10.1371/journal.ppat.1000165

**Published:** 2008-09-26

**Authors:** Desigan Kumaran, Richa Rawat, S. Ashraf Ahmed, Subramanyam Swaminathan

**Affiliations:** 1 Biology Department, Brookhaven National Laboratory, Upton, New York, United States of America; 2 Integrated Toxicology Division, US Army Research Institute of Infectious Diseases, Fort Detrick, Maryland, United States of America; The Rockefeller University, United States of America

## Abstract

The seven antigenically distinct serotypes of *Clostridium botulinum* neurotoxins, the causative agents of botulism, block the neurotransmitter release by specifically cleaving one of the three SNARE proteins and induce flaccid paralysis. The Centers for Disease Control and Prevention (CDC) has declared them as Category A biowarfare agents. The most potent among them, botulinum neurotoxin type A (BoNT/A), cleaves its substrate synaptosome-associated protein of 25 kDa (SNAP-25). An efficient drug for botulism can be developed only with the knowledge of interactions between the substrate and enzyme at the active site. Here, we report the crystal structures of the catalytic domain of BoNT/A with its uncleavable SNAP-25 peptide ^197^QRATKM^202^ and its variant ^197^RRATKM^202^ to 1.5 Å and 1.6 Å, respectively. This is the first time the structure of an uncleavable substrate bound to an active botulinum neurotoxin is reported and it has helped in unequivocally defining S1 to S5′ sites. These substrate peptides make interactions with the enzyme predominantly by the residues from 160, 200, 250 and 370 loops. Most notably, the amino nitrogen and carbonyl oxygen of P1 residue (Gln197) chelate the zinc ion and replace the nucleophilic water. The P1′-Arg198, occupies the S1′ site formed by Arg363, Thr220, Asp370, Thr215, Ile161, Phe163 and Phe194. The S2′ subsite is formed by Arg363, Asn368 and Asp370, while S3′ subsite is formed by Tyr251, Leu256, Val258, Tyr366, Phe369 and Asn388. P4′-Lys201 makes hydrogen bond with Gln162. P5′-Met202 binds in the hydrophobic pocket formed by the residues from the 250 and 200 loop. Knowledge of interactions between the enzyme and substrate peptide from these complex structures should form the basis for design of potent inhibitors for this neurotoxin.

## Author Summary

Botulinum neurotoxins are the most poisonous substance to humans. The ease with which the bacteria can be grown, its potency and persistence have made it a potential bioterrorism agent, and accordingly, botulinum neurotoxin has been declared as Category A agent by the Centers of Disease Control and Prevention. Since it is both a potential bioweapon and a bioterrorism agent, it is imperative to develop counter measures and therapeutics for these neurotoxins, as none are available so far except experimental vaccines and an FDA-approved equine antitoxin. Our work presented here is an important milestone towards achieving this goal. The best antidote can be developed by blocking the active site of any enzyme. The crystal structures of substrate peptide–enzyme complex presented here map the interactions between the two and provide critical information for designing effective drugs against this toxin.

## Introduction

Clostridium botulinum neurotoxins (CNTs) are the most potent toxins known to humans since even one billionth of an ounce is fatal. Seven antigenically distinct botulinum neurotoxins are produced by the bacterium *Clostridium botulinum* and they share considerable sequence homology, and structural and functional similarity [1]–[3]. They are produced as inactive single chains of molecular mass 150 kDa and released as active dichains, a heavy chain (HC, 100 kDa) and a light chain (LC, 50 kDa) held together by an interchain disulfide bond [4]–[7]. HC comprising two distinct domains is responsible for binding to neuronal cells and translocation into cytosol. LC is the catalytic domain cleaving one of the three proteins forming the SNARE complex (Soluble N-ethylmaleimide-sensitive fusion protein attachment protein receptors) required for docking and fusion of vesicles containing neurotransmitters to target cells [8]–[12]. The SNARE complex formation is prevented when any of the SNARE proteins is cleaved and accordingly blocks neurotransmitter release leading to flaccid paralysis and eventual death.

Catalytic domains of BoNTs are zinc proteases and cleave SNARE proteins with stringent substrate specificity though they share significant sequence similarity. BoNT/A and BoNT/E cleave the synaptosomal-associated 25 kDa protein (SNAP-25) while BoNT/B, /D, /F, and /G cleave the vesicle-associated membrane protein (VAMP). BoNT/C is the only one that has dual substrate specificity, *viz* SNAP-25 and syntaxin [13]. The enhanced substrate specificity of CNTs is due to the recognition of substrates at remote sites called exosites in addition to the active site [14].

The potency and the ease with which these toxins can be produced make them potential bioweapons and bioterrorism agents. The Centers for Disease Control and Prevention (CDC) has declared them as Category A biowarfare agents. Currently, while experimental vaccines are available, only an equine trivalent antitoxin is available for post-exposure therapeutics with a limited therapeutic window [15]. One of the most effective ways a drug can act is by blocking the site where the substrate binds to toxin and accordingly the crystal structure of substrate-enzyme complex is essential to map out a strategy. Even though crystal structure of SNAP peptide (146–206)-inactive enzyme complex is available, it lacks interactions at the active site since the enzyme used was an inactive double mutant [14]. Here we present for the first time the structure of the substrate peptide, QRATKM containing the scissile peptide bond, bound to the active enzyme. This crystal structure reveals interesting features of complex formation which can help in designing efficient drug molecules to prevent or treat botulism. It is remarkable that this natural substrate peptide is not cleaved by the enzyme. In addition, we are also reporting the crystal structure of RRATKM, a variant of the substrate peptide, in complex with the enzyme. Though both are weak inhibitors, RRATKM is a better inhibitor than QRATKM.

## Materials and Methods

### Protein expression and purification

*Clostridium botulinum* neurotoxin serotype A truncated light chain (residues 1 to 424), Balc424, was expressed in *E. coli* and purified to homogeneity using size exclusion chromatography, as described previously [16]. The purified enzyme in 20 mM HEPES, 2 mM DTT, 200 mM NaCl, pH 7.4 was stored at −20°C until used. Amides of the peptides, QRATKM and RRATKM, were custom synthesized by Peptide 2.0 Inc., Chantilly, VA20153, USA. The stock solutions of the peptides were prepared with the above mentioned buffer.

### Crystallization and data collection

Balc424-QRATKM and Balc424-RRATKM complex crystals were grown using a range of protein/peptide molar ratio (1∶5 to 1∶30). Both QRATKM and RRATKM complex crystals were grown by sitting drop vapor diffusion at room temperature. Briefly, 3 µl of the protein solution (15 mg/ml) was mixed with an equal volume of a reservoir solution containing 20% PEG 8000, 100 mM sodium cacodylate, pH 6.5, 5% ethylene glycol and 200 mM ammonium sulfate. Thick plate-like crystals were obtained in five days and were flash frozen with liquid nitrogen using 20% ethylene glycol as cryoprotectant. The X-ray intensity data for both complex crystals were collected at X29 beamline of National Synchrotron Light Source (NSLS) using ADSC QUANTUM 315 detector. Balc424-QRATKM and Balc424-RRATKM complex crystals diffracted to 1.5 Å and 1.6 Å, respectively and belonged to the *P*2_1_ space group with one molecule in the asymmetric unit (Table 1). All data were processed using the HKL2000 suite [17].

**Table 1:** Crystal data and refinement statistics of Balc424 with substrate peptide complexes

Name/code		QRATKM	RRATKM
Cell dimensions	a (Å)	49.14	50.87
	b	66.20	66.58
	c	64.82	65.06
	β (°)	99.10	98.3
Space group		P2_1_	P2_1_
Resolution range (Å)	Overall	50–1.5	50–1.6
	Last shell	1.54–1.50	1.65–1.6
# unique reflections		64,562	53,272
Completeness (%)			
	(Overall/Last shell)	97.4/80.0	95.4/76.3
R_merge_ 1 overall/last shell		0.066/0.25	0.059/0.15
<I/σ(I)> overall/last shell		17/2.0	23.0/3.0
**Refinement Statistics**			
Resolution (Å)		50–1.5	50–1.6
R factor^2^/Rfree (%)		18.0/20.0	20.0/22.0
R.M.S deviation from ideality			
	Bond lengths (Å)	0.005	0.005
	Bond angles (°)	1.2	1.2
Average B-factors (Å^2^)			
	Main chain	14.0	21.1
	Side chain	16.2	23.0
	Waters	22.6	29.7
	Ions	27.0	35.3
	Substrate peptide	31.0	28.4
Number of atoms			
	Proteins	3,423	3,423
	Waters	375	375
	Ions (Zn^2+^/SO_4_^2−^)	1/5	1/10
	Ligands	50	52
Surface area Å^2^(total/buried)		993/726	1023/739
(by substrate peptide)			
Residues (%) in the core			
region of φ-ψ plot		91.2	89.0

1 R_merge_ = ∑_j_(|I_h_−<I>_h_|)/∑I_h,_ where <I_h_> is the average intensity over symmetry equivalents

2 R-factor = ∑|F_obs_−F_calc_|/∑|F_obs_|

### Structure determination

The structures of the complexes were determined by Fourier Synthesis using the acetate bound Balc424 (Protein Data Bank id 3BWI) as model followed by rigid-body refinement and simulated annealing. The composite omit map and the difference Fourier showed interpretable electron density for these hexapeptides. The best results were obtained with data collected from crystals grown with 1∶25 (protein/peptide) molar ratio. The peptide models were built with O [18] and further refined with CNS [19] until convergence. The final refinement statistics are shown in Table 1. Models were validated with the Ramachandran plot using PROCHECK [20].

### Activity assay

The proteolytic activity of balc424 was determined by HPLC using P[187–203] synthetic peptide as reported previously [21]; [22]. Briefly, balc424 enzyme (550 nM) was incubated with the 17-mer peptide (1mM) at 37°C for 30 min in the assay buffer (50 mM HEPES, 0.25 mM ZnCl_2_, 5.0 mM DTT, pH 7.2). *IC_50_* values were determined by varying the concentration of inhibitors. The experimental data were analyzed using equation 1, where *I* is the inhibitor concentration, *y* is the percent inhibition, with a slope factor (s) of 1.0.
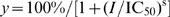
(1)


Coordinates and structure factors have been deposited to the Protein Data Bank. BALC424-QRATKM (3DDA) and BALC424-RRATKM (3DDB). The SwissProt accession number for BoNT/A is P10845.

## Results

### Crystal structure of Balc424 with QRATKM

**Figure 1:**
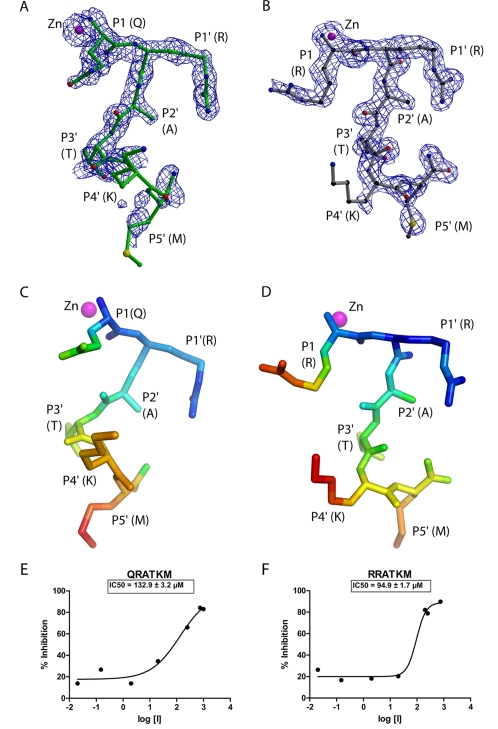
Hexapeptides and inhibition plots. Electron density maps (blue mesh) for bound substrate (QRATKM) and its variant (RRATKM) are shown in A and B, respectively. The electron density is from composite omit maps (2|F_o_|-|F_c_|) and contoured at 1σ level. QRATKM (green) and RRATKM (gray) peptides are shown in ball and stick model. Zinc, oxygen and nitrogen atoms are shown as magenta, red and blue spheres, respectively. Carbon atoms are shown in green (QRATKM) and black (RRATKM). Figures were prepared using Molscript, Raster3D and Bobscript [34]–[36]. Distribution of B factors for the QRATKM and RRATKM are shown in C and D, respectively. The peptide atoms are colored according to B factor with RGB (Red-Green-Blue) color ramp with blue and red corresponding to the lowest (17 Å^2^) and highest (50 Å^2^). Pymol (DeLano, W.L. The PyMOL Molecular Graphics System (2002) on World Wide Web http://www.pymol.org) was used to prepare C and D figures. Inhibition of Balc424 catalytic activity at increasing concentrations (µM) of QRATKML (E) and of RRATKML (F).

**Figure 2:**
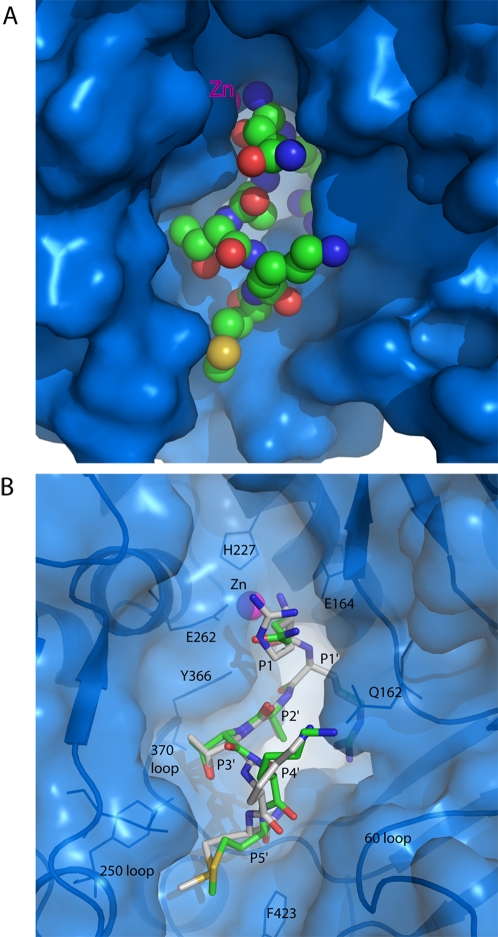
Binding of the substrate peptide in the active site of Balc424. (A) Figure shows the substrate peptide (QRATKM) binding in the active site of Balc424. Balc424 is shown in solid blue-colored surface. Substrate peptide is shown in sphere (CPK) model. Carbon, oxygen, nitrogen, sulfur and zinc atoms are shown in green, red, blue, yellow and magenta, respectively. (B). Superposed stick models of QRATKM and RRATKM are shown in green, and gray, respectively. Carbon atoms are shown in green (QRATKM) and gray (RRATKM). Balc424 is shown in semi-transparent surface (blue) representation with secondary elements at the active site pocket. Only a few substrate-binding residues are shown as markers. Pymol (DeLano, W.L. The PyMOL Molecular Graphics System (2002) on World Wide Web http://www.pymol.org) was used to prepare these figures.

**Figure 3:**
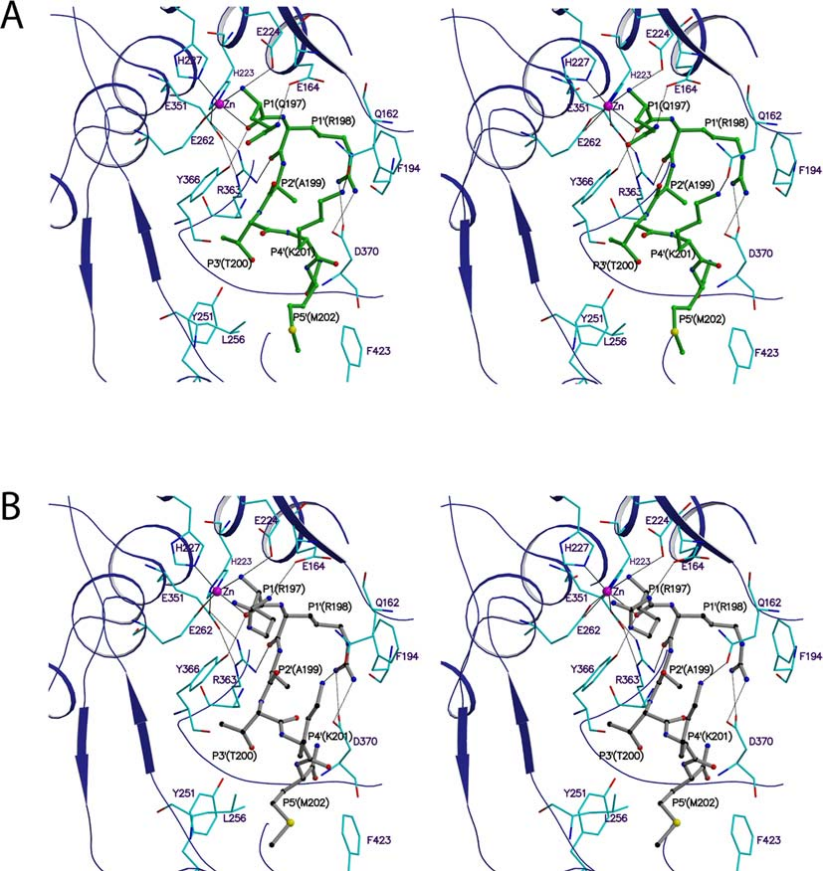
Structure of Balc424 in complex with the QRATKM, a segment of substrate SNAP-25. (A). Stereo view of the active site shows the molecular interactions between the substrate peptide (QRATKM) and protease (Balc424). Protease and substrate residues are shown in cyan and green, respectively. Blue color ribbon represents the protease secondary elements at the vicinity of the active site. (B). Stereo view of the active site center of Balc424 (cyan stick) with RRATKM (gray ball and stick). Oxygen, nitrogen, sulfur and zinc atoms are shown in red, blue, yellow and magenta, respectively. Hydrogen bonds are depicted as black dash lines while zinc co-ordination is shown in solid line.

The crystal structure has been determined to 1.5Å resolution. The model refined with R and R free of 18.4 and 20.1%, respectively. The final refined model contains 423 protease residues, 6 substrate residues, one sulfate and one zinc ions and 375 waters. More than 91% of residues are within the most allowed region of the Ramachandran plot. The electron density in the residual map (Fo-Fc) was well defined for the hexapeptide and QRATKM could be modeled unambiguously except for the side chains of K and M (Figure 1A). It appears that K could take two rotamer positions. This is the first time an uncleavable substrate bound structure of an active botulinum neurotoxin has been reported and it has helped in unequivocally defining S1 to S5′ sites. Most notably, the amino nitrogen and carbonyl oxygen of P1 residue (Gln197) chelate the zinc ion (Figures 2 and 3). The amino nitrogen has replaced the nucleophilic water as was shown earlier [16].

### Crystal structure of Balc424 with RRATKM

**Figure 4:**
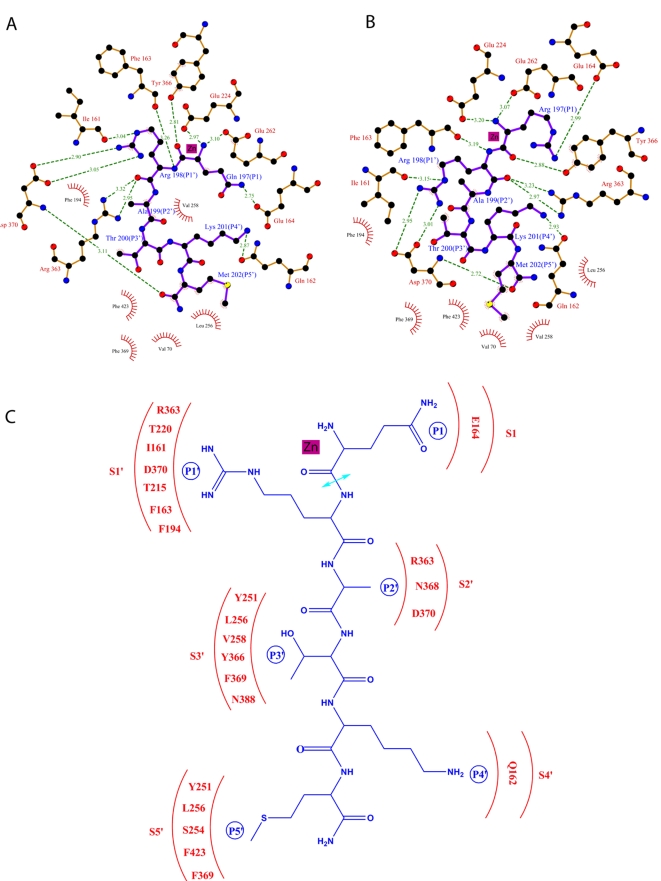
Schematic diagram of the molecular interactions between the Balc424 and substrate peptides. The interactions between Balc424 active site residues and the substrate peptides are shown. (A) QRATKM and (B) RRATKM. Black, red, blue, and yellow colored circles represent carbon, oxygen, nitrogen, and sulfur atoms, respectively. For clarity, zinc co-ordination and water molecules involved in the interactions at the active site are not shown. (C). A Schematic diagram representing S1 to S5′sites. Residues of the enzyme forming the subsites and substrate peptide are shown in red and blue, respectively. Proteolytic site is shown in cyan colored double-headed arrow. Figures A and B were prepared with Ligplot [37]. ChemDraw ultra (CambridgeSoft, Inc) was used to prepare figure C.

The crystal structure of Balc424 with a substrate analog RRATKM has been determined to 1.6Å resolution. The R and R free for the final refined model are 20.1 and 21.2%, respectively. The final refined model contains 423 residues of protease, 6 residues of substrate analog peptide, two sulfate ions, one zinc ion and 375 waters. More than 90% of residues are within the most allowed region of the Ramachandran plot. The substrate analog could be modeled unambiguously in the residual map (Fo-Fc) (Figure 1B). Except for some minor variations of side chain orientations, the hexapetide RRATKM binds similar to the substrate peptide QRATKM (Figures 2, 3 and 4). As in the case of QRATKM, the P1 (Arg197) amino group and the carbonyl oxygen chelate the catalytic zinc and the nucleophilic water has been replaced. P1-P5′ residues occupy identical subsites as in QRATKM. This kind of interaction seems to be common with all peptide analog inhibitors [16] and probably plays a dominant role in inhibiting the catalytic activity.

Though we have shown earlier that short tetrapeptides (analogs of substrate) are good inhibitors (nM range), the hexapeptides are weak inhibitors [16]. The *IC_50_* of QRATKM and RRATKM are 133 and 95 µM, respectively (Figures 1E and 1F).

## Discussion

### Mapping of S1–S5′ subsites

The side chain of P1-Q197 is exposed to the solvent region but makes a hydrogen bond with Glu164 OE1 (Figures 3 and 4). However, it is stabilized by various other interactions as well. N and O chelate zinc while O is also hydrogen bonded to Tyr366 OH which stabilizes the substrate and positions it for catalytic activity. Mutation of Tyr366 to Phe or Ala resulted in dramatic decrease in activity [23]; [24]. The amino nitrogen which has replaced the nucleophilic water is hydrogen bonded to Glu224 OE1 and OE2 (the latter through a water molecule). It is known that variation in P1 does not affect the catalytic activity, probably due to most of the interactions being with the main chain atoms [25]–[28]. Mutation of Glu164 to Gln only had a marginal effect on the catalytic activity [23]. The only difference between QRATKM and RRATKM is at P1 residue. This was based on our previous experience with tetrapeptides [16] since the positive charge on Arg197 better complements the charge in the active site cavity. While P1-Gln197 makes a hydrogen bond with Glu164, P1-Arg197 makes a salt bridge interaction with Glu164 thus making it more strongly bound (Figures 3 and 4). There are additional interactions with a sulfate ion nearby but this may be an artifact of crystallization. Other than this, residues from 198 to 202 in both structures superpose well except for minor variations in side chain orientations (Figure 2B). The following discussion on subsites S1′ to S5′ applies equally for the both structures.

P1′-Arg198 occupies the S1′ site formed by Arg363, Thr220, Asp370, Thr215, Ile161 and Phe194. Phe163, though slightly farther, also forms part of this subsite. The amino nitrogen and carbonyl oxygen of P1′ are hydrogen bonded to Phe163 O and Arg363 NH2 (Figures 3 and 4). These two interactions stabilize the substrate binding. When Arg363 is mutated to Leu or Ala, the activity decreases by 620 and ∼80 fold, respectively [23]; [24]. In addition, the guanidinium group of P1′ Arg198 forms salt bridges with Asp370 and P1′-Arg198 NE forms a hydrogen bond with Ile161 O. The salt bridge interaction between P1′-Arg198 and Asp370 is crucial since mutation of Asp370 reduced the catalytic activity by 250–600 fold [23]; [29]. The other major interaction is the stacking of guanidinium group of P1′-Arg198 with Phe194 (Figure 3). This stacking interaction also plays a major role in the activity since Balc424 Phe194Ala has ∼100 fold less activity [29]. Accordingly, both the electrostatic and hydrophobic interactions are crucial for catalytic activity. The S1′ site is fairly big and gives enough flexibility for Arg198. In substrate analog tetrapeptide inhibitor complexes, it takes various rotamer positions [16]. In BoNT/A arginine hydroxamate complex structure, Arg hydroxamate occupies the S1′ site. But Zn is chelated by the carbonyl oxygen and the hydroxamate group. Also the direction of the peptide N to C is reversed [30].

S2′ site is formed by Arg363, Asn368 and Asp370, while S3′ subsite is formed by Tyr251, Leu256, Val258, Tyr366, Phe369 and Asn388. P3′-Thr200 OG makes a hydrogen bond with Tyr251 OH. P4′-Lys201 is exposed to the solvent region. In the present crystal structure the side chain density for this residue is weak probably due to high thermal factors (Figures 1C and 1D). However, one of the rotamer positions could form a hydrogen bond with Gln162 OE1. This does not form a hydrogen bond in the complex structure of BoNT/A-SNAP-25 (146–206) (PDB id = 1XTG). Instead Glu257 is close by, about 4.5Å. S5′ site is made of Tyr251, Phe369, Leu256, Ser254 and Phe 423. P5′-Met202 occupies this hydrophobic pocket (Figure 4C).

### Comparison of SNAP-25(146–206) and QRATKM at the active site

**Figure 5:**
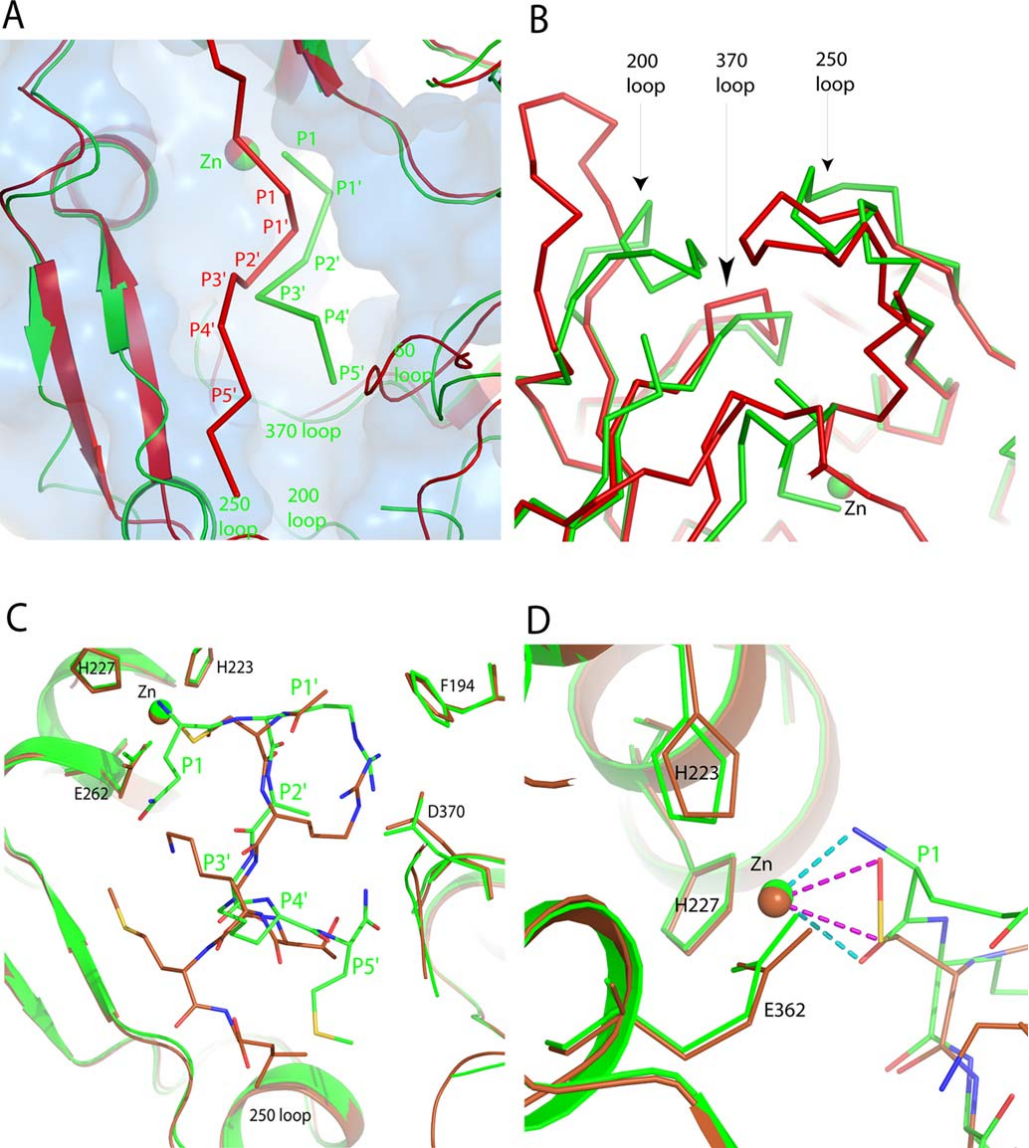
Comparison of Balc424 complexed with segments of SNAP-25 (197–202) and (146–204) and an inhibitor (N-Ac-CRATKML). (A) and (B) show the superposition of Balc424 with SNAP-25 (197–202) and (146–204). Green and red represent SNAP-25 (197–202) and SNAP-25 (146–204), respectively. Superposition of Balc424 with substrate peptide (SNAP-25 [197–202]) and N-Ac-CRATKML peptide at the vicinity of the active site are shown in C and D. N-Ac-CRATKML peptide complex is shown in brown color. Zinc binding and selective substrate binding residues are in stick model.

The crystal structure of SNAP-25 (146–206) peptide with an inactive double mutant (Pdb id = 1XTG) had identified the exosites as recognition sites distant from the active site [14]. However, the region of SNAP-25 peptide near the active site was disordered and could not be modeled very well. Comparison of the C-alpha position of the corresponding residues in the present structure shows that the C-alpha positions of these six residues are shifted. C-alphas of 197, 198, 199, 200, 201 and 202 are 4.34, 3.84, 3.55, 3.13, 5.69 and 6.12Å for the corresponding C-alphas in the present structures (Figure 5A). In the absence of Tyr366, SNAP25 residues near the active site move towards 250 loop increasing the distance from catalytic zinc. When the wild type light chain is used, the SNAP peptide is closer to the catalytic zinc and the 170 loop. This shift is probably due to either the disorder or the inactive mutant in 1XTG. One possibility is that since residues corresponding to α-exosites are missing in the short peptide, the whole peptide could have slid down. But this possibility is less likely since the β-exosite interaction is maintained in both the structures. Though the C-alpha atom of P5′ in the current structure and 1XTG are farther apart, the side chains occupy the same place. We conclude that this shift is due to the loss of interaction of SNAP-25 with Tyr366 which has been mutated to Phe in 1XTG. Because of this difference, P4′-Lys201 has potential interaction with Gln162 of the enzyme rather than Glu257. The length of the anti-parallel β sheet formed near the 250 loop (β-exosite) in 1XTG (13 Å) is almost double the length as in QRATKM (6.5 Å) (Figure 5A). Based on the above observations, the subsites as identified in this structure truly represent the substrate-enzyme complex interactions.

Though the overall conformation of the enzyme in 1XTG and the current structure is very similar (RMSD is ∼1 Å for 400 Cα atoms), loops 200 and 250 vary significantly (Figure 5B). This conformational change may be either due to the recognition of α-exosites in 1XTG or just an artifact of crystal packing. In the current structure, loops 200, 250 and 370 pack together tightly whereas in the 1XTG, 200 loop moved away. The C-alphas of Pro206 (within 200 loop) in 1XTG and QRATKM complex are ∼12 Å apart.

### Comparison of QRATKM with N-Ac-CRATKML

Recently, the structure of a complex between the BoNT/A-LC and an inhibitory peptide N-Ac-CRATKML has been reported [31]. Though the direction of the polypeptide is the same, the inhibitory peptide (N-Ac-CRATKML) is shifted down by one residue compared to the substrate peptide QRATKM (Figure 5C). This appears to be due to the effect of oxidation of Cys and the N-terminal blocking acetyl group. The cysteine is oxidized to sulfenic form. Both the sulfur and the OH group chelate the zinc ion unlike in QRATKM complex where the carbonyl oxygen and amino nitrogen of P1 residue chelate zinc (Figure 5D). As a consequence, the acetyl group takes the C-alpha position of P1′ (Arg198) and P1′ arginine moves to P2′ alanine's place. Moreover, P1 carbonyl oxygen interacts with Arg363 instead of Tyr366. In QRATKM, P1' arginine forms salt bridge with Asp370 through guanidinium:carboxylate pair whereas in the N-Ac-CRATKML it is through a single NE and OD1 interaction. Interestingly, even though the C-alpha position has moved, Arg198 side chain takes a different rotamer position made possible by the size of the cavity and stays in the same pocket. In addition, P4' lysine interacts with Tyr366 while in the substrate peptide (QRATKM) it interacts with Glu162. Hence the positioning of the inhibitory peptide (N-Ac-CRATKML) may not represent the substrate binding position as in QRATKM structure. In both cases the enzyme does not undergo significant conformational changes as it did in the structure of SNAP-25 (146–206) peptide complex [14].

### Roles of substrate amino acid residues spanning the cleavage site

N-Ac-CRATKML is a fairly good inhibitor (*Kι* 1.9 µM) [28]. But when the N terminal Cys is replaced with 2-mercapto-3-phenylpropionyl (mpp) the *Ki* improved to 300nM. Keeping this as a control various truncations were done [27]. Truncating the last three residues of the mpp derivative (KML) increased the Ki 100-fold while deletion of only the last two increased it only by ∼13-fold. The importance of Lys201 of the substrate may be attributed to the potential hydrogen bond the terminal side chain atom (NZ) makes with Gln162. Mutation of Lys201 to Ala increased the Ki 10 fold suggesting that the Lys side chain interaction is crucial. When Thr200 of the substrate was mutated to Ala, Ki increased only marginally since the hydrogen bond with OG was lost. However, it is not clear from the present structure why Ala199Val will increase the Ki <10 fold. A simple modeling shows that the S2' subsite is big enough to accommodate a Val. Mutation of Arg198 to Lys increases Ki by more than 1000 fold. This is because both the salt bridge and stacking interactions are lost. It appears stacking may be important since ionic interaction between Lys201 and Asp370 is still possible. Though the present hexapeptide lacks Leu203, truncation of this peptide had no effect on Ki.

### Recognition and binding of substrate by Balc424

Saturation mutation studies based on the crystal structure of BoNT/A with SNAP-25 (146–206) has been used to define two regions, active site (AS) domain and binding site (B) domain in SNAP-25 [14]; [29]. SNAP-25 residues 193–202 form AS while residues 156–181 form B. Our hexapeptides form part of AS only. In the same work, two minimal length peptides have been tested for catalytic activity, D^193^EANQRATK^201^ (SN/A1) and A^195^NQRATK^201^ (SN/A2) (the numbers correspond to our numbering scheme). While SN/A1 was cleavable by BoNT/A, SN/A2 was not, suggesting that the N terminal DEAN is required for cleavage. This probably explains why QRATKM which lacks DEAN was not cleaved in our case even though we used up to 1∶30 ratio of Balc424 to peptide. However, the major reason for the peptide not being cleaved is the amino group chelating zinc. Any extension beyond in the N terminal direction would change the character of this amino group and may not be able to chelate zinc. However, the earlier study used GST fusion protein to express the short peptide and might have some effect in binding to the enzyme. This is supported by the facts that I^192^DEANQRATKKMLGSG^207^ had 1/5^th^ the activity compared to wild type [22] and the mutants A195C and N196C in the 17-mer SNAP-25 substrate peptide [28] insignificantly affected *K_m_* and *k_cat_*.

The current structure confirms our earlier model for catalytic mechanism [16]. Glu224 acts as the general base in abstracting a proton from the nucleophilic water and also helps in shuttling protons to the leaving group. In addition, the roles of Arg363 and Tyr366 are to stabilize the substrate for proper positioning and orientation as the carbonyl oxygens of P1 and P1' are hydrogen bonded to Tyr366 and Arg363. Tyr366 further stabilizes the oxyanion role of P1 carbonyl oxygen. Another molecular mechanism for BoNT/A recognition and cleavage of SNAP-25 has been proposed [29]. In that mechanism P5 (Asp193) residue of SNAP25 is supposed to make the initial contact with the enzyme at the α-exosites by forming a salt bridge with Arg177. This in turn aligns P4'-Lys201 to form a salt bridge with Glu257. These interactions are supposed to broaden the active site and allow P1'-Arg198 to dock into the S1' site by both electrostatic and hydrophobic interactions. The current structure does not support such a mechanism. First, the substrate peptide is able to dock into S1' site even though the peptide lacks substrate residues upstream of P1. Second, the S1' site of Balc424 with and without bound peptide is similar and there is no indication of any change in shape or size. Third, there is no possibility for Lys201 to make hydrogen bond contact with Glu257. Accordingly, our crystallographic data show that Balc424 is well positioned for peptide binding and catalytic action without having to undergo a conformational change. However, the interaction of P4' with S4' substrate may be disrupted after cleavage and help the substrate to leave allowing uncleaved peptide to bind in its place. But there is no experimental or mutational evidence for that.

### Implication for drug design

Even though botulinum neurotoxins are declared category A biowarfare agents, effective drugs are yet to be developed. Antibody therapeutics is emerging but more than one antibody may be needed to contain the effect of a single serotype [32]. An equine antitoxin is also available for post exposure therapeutics. Small molecule inhibitors are being developed but the active site of botulinum neurotoxin is large and it would be better to have larger molecules or strongly binding peptidomimetic inhibitors to block the active site. The current structure where S1 to S5' sites have been mapped unequivocally will be a good starting point. This would at least give a serotype specific inhibitor that could be transformed into an effective drug for botulinum neurotoxin A. We have shown that the P1 residue could be changed to Arg without affecting the binding efficiency and in fact it has proved to be a better inhibitor since it complements the charge in that region. It is known that changing it to cysteine improves binding [27]. However, oxidation of Cys may cause a problem. The structural environment of P1 also suggests that an amino acid containing an aromatic ring may be better suited as it would improve stacking interactions. The hexapeptide could be extended by one residue at the N terminus. However, it might affect the chelation of zinc by P1 amino group. The requirement of P1' Arg is crucial for BoNT/A activity. However, changing it to Tyr will still keep the stacking interaction though the salt bridge would be lost. Arg198Ala abolishes the activity without affecting the Km value [33]. S2' site also suggests that it can tolerate bigger hydrophobic, aromatic residue. It is possible to introduce modifications in the peptides to bring rigidity, specificity and resistance from proteases. There are endless possibilities that can be tried with the information provided by this structure. Our biochemical assays with full length and truncated balc (balc424) do not show much variation and hence the results are equally applicable to both. It is desirable to have a broad spectrum inhibitor to be effective across the serotypes and this structure will be a starting point.
